# Vector competence of *Aedes aegypti*, *Aedes albopictus*, and *Culex quinquefasciatus* mosquitoes for Mayaro virus

**DOI:** 10.1371/journal.pntd.0007518

**Published:** 2020-04-14

**Authors:** Thiago Nunes Pereira, Fabiano Duarte Carvalho, Silvana Faria De Mendonça, Marcele Neves Rocha, Luciano Andrade Moreira

**Affiliations:** Grupo Mosquitos Vetores: Endossimbiontes e Interação Patógeno-Vetor, Instituto René Rachou—Fiocruz, Belo Horizonte, MG, Brazil; Faculty of Science, Mahidol University, THAILAND

## Abstract

Newly emerging or re-emerging arthropod-borne viruses (arboviruses) are important causes of human morbidity and mortality worldwide. Arboviruses such as Dengue (DENV), Zika (ZIKV), Chikungunya (CHIKV), and West Nile virus (WNV) have undergone extensive geographic expansion in the tropical and sub-tropical regions of the world. In the Americas the main vectors of DENV, ZIKV, and CHIKV are mosquito species adapted to urban environments, namely *Aedes aegy*pti and *Aedes albopictus*, whereas the main vector of WNV is *Culex quinquefasciatus*. Given the widespread distribution in the Americas and high permissiveness to arbovirus infection, these mosquito species may play a key role in the epidemiology of other arboviruses normally associated with sylvatic vectors. Here, we test this hypothesis by determining the vector competence of *Ae*. *aegypti*, *Ae*. *albopictus*, and *Cx*. *quinquefasciatus* to Mayaro (MAYV) virus, a sylvatic arbovirus transmitted mainly by *Haemagogus janthinomys* that has been causing an increasing number of outbreaks in South America, namely in Brazil. Using field mosquitoes from Brazil, female mosquitoes were experimentally infected, and their competence for infection and transmission rates of MAYV was evaluated. We found consistent infection rate for MAYV in *Ae*. *aegypti* (57.5%) and *Ae*. *albopictus* (61.6%), whereas very low rates were obtained for *Cx*. *quinquefasciatus* (2.5%). Concordantly, we observed high potential transmission ability in *Ae*. *aegypti* and *Ae*. *albopictus* (69.5% and 71.1% respectively), in contrast to *Cx*. *quinquefasciatus*, which could not transmit the MAYV. Notably, we found that very low quantities of virus present in the saliva (undetectable by RT-qPCR) were sufficiently virulent to guarantee transmission. Although *Ae*. *aegypti* and *Ae*. *albopictus* mosquitoes are not the main vectors for MAYV, our studies suggest that these mosquitoes could play a significant role in the transmission of this arbovirus, since both species showed significant vector competence for MAYV (Genotype D), under laboratory conditions.

## Author summary

The present study demonstrated that *Ae*. *aegypti* and *Ae*. *albopictus* mosquitoes can be competent laboratory vectors for MAYV. In contrast, *Cx*. *quinquefasciatus* mosquitoes were refractory to MAYV. Regarding the viral dilution and nanoinjection, a higher detection sensitivity was observed after virus nanoinjection into naïve mosquitoes, indicating that only a few viral particles are required to infect mosquitoes, and these particles may not be detected by RT-qPCR before the nanoinjection procedure.

## Introduction

The mosquitoes *Ae*. *aegypti*, *Ae*. *albopictus*, and *Cx*. *quinquefasciatus* are widely distributed throughout the world, especially in tropical and subtropical regions [1–3]. They are considered a serious concern to public health as vectors of several arboviruses such as DENV, ZIKV, CHIKV, and WNV [4–13]. Studies have shown that *Ae*. *aegypti* and *Ae*. *albopictus* mosquitoes exhibit laboratory vector competence for the infection and transmission of MAYV, and *Cx*. *quinquefasciatus* mosquitoes infected with MAYV have been found in Cuiabá [14–16].

The MAYV was first isolated in 1954 from rural workers in Mayaro, on the island of Trinidad [17] and, like CHIKV, it is an arbovirus of the genus *Alphavirus*, belonging to the family *Togaviridae* [18–20]. To date, three MAYV genotypes (D, L and N) have been identified. The D genotype has a wide geographical distribution, occurring in Brazil, Bolivia, Peru, Suriname, Trinidad, Tobago, Argentina, Colombia, and Venezuela [21–25]. The L genotype was isolated in Brazil and Haiti [24] and contains strains detected only in Brazil[26]. On the other hand, the N genotype, was discovered in an outbreak in 2015 in Venezuela [27].

The MAYV is transmitted primarily by the bite of female mosquitoes of the genus *Hemagogus*, and in nature several vertebrates can host this virus, which is detected in non-human primates, rodents, birds, sloths, and other small mammals [28].

In humans, MAYV infection usually occurs in people with a history of activities in forested areas [18–21]. Long *et al*. [14], described, in febrile humans, a high load of viral RNA, determined by real-time polymerase chain reaction, ranging from 5.01×10^2^ to 2.18×10^5^ (log_10_/PFU equivalents/mL). This disease has similar symptomatology to DENV and/or CHIKV, causing an acute and self-limiting febrile illness, which may be accompanied by hemorrhagic phenomena such as petechiae and gingival bleeding. Usually the wrist, ankle, hands, and feet joints are significantly affected, and symptoms may persist for several months, incapacitating the infected person [22,23,29]. In recent years in Brazil, several cases of MAYV have been registered in Pará (2008), Mato Grosso (2012), and Goiás (2014–2016) [20–22,30–34]. Although there is still no evidence of the transmission efficiency of MAYV in an urban cycle, it has the potential to establish an epidemic scenario in the Americas, similar to what occurred with ZIKV and CHIKV [35]. Using mathematical models taking into account outbreaks since 1960 and increasing global temperature, Lorenz *et al*. [36] predicted that MAYV would expand its area of ​​coverage in the coming years. Thus, the importance of MAYV as a human pathogen with the potential to emerge in urban areas is strong. Therefore, the main objective of this study was to evaluate the vector competence of *Ae*. *aegypti*, *Ae*. *albopictus*, and *Cx*. *quinquefasciatus* for MAYV, since these mosquitoes may be involved in the dispersion of this virus.

## Materials and methods

### Ethics statement

The human blood used in all experiments was obtained from a blood bank (Fundação Hemominas), according to the terms of an agreement with the Instituto René Rachou, Fiocruz/MG (OF.GPO/CCO agreement—Nr 224/16).

### Mosquito species and rearing

For this study, three mosquito species were used: *Ae*. *aegypti*, *Ae*. *albopictus*, and *Cx*. *quinquefasciatus*. *Ae*. *aegypti* and *Ae*. *albopictus* mosquitoes were collected from ovitraps, whereas *Cx*. *quinquefasciatus* were collected using an entomological ladle. All collections occurred in the neighborhood of Pampulha (19.8527° S, 43.9560° W), in the city of Belo Horizonte, Brazil, in the first half of 2017. The *Ae*. *aegypti* and *Ae*. *albopictus* populations were initiated from at least 2,000 eggs for each species, whereas for *Cx*. *quinquefasciatus*, the colony was obtained with more than 3,000 larvae.

Eggs/larvae were transported to the insectary of the René Rachou Institute, Fiocruz/MG, and were kept in a controlled environment, at 27 ± 2°C and ∼82% RH with a 12:12 h light/dark cycle. Larvae were maintained with fish food pellets, Tetramin tropical and GoldFish Colour. After emergence and identification, adults were kept on a 10% sucrose solution regimen, *ad libitum*. Adult females were fed with human blood in an artificial feeder for egg production. To simulate field conditions and minimize the effects of inbreeding and population colonization, all experiments were conducted with mosquitoes from the same geographic area and up to the third generation.

### Mayaro virus culture

MAYV isolated from the serum of an infected human in Trinidad (strain TRVL 4675) in 1954 and belonging to the D genotype was originally acquired from the American Type Culture Collection (ATCC). Full-length sequences are available on GenBank [37]. To prepare the MAYV for oral feeding, a frozen virus stock (an aliquot of MAYV was kindly supplied by the Flavivirus Laboratory of the Oswaldo Cruz Instituto—IOC / Fiocruz) was passaged once through C6/36 cells (approximately 2 million cells) in Leibowitz L-15 medium supplemented with 10% fetal calf serum and maintained at 28°C. The supernatant viral stock was harvested after day 4 (for the second experiment and at 5 days for the first experiment). Both viral titers were quantified after a freeze-thaw cycle and two replicates (A and B) were used to evaluate the mosquito infection rates. For the first replicate, the viral titer was 1×10^9^ PFU/mL, and for the second, 6×10^9^ PFU/mL.

### Mosquito infection and transmission analysis

In order to analyze infection rates and transmission rates of MAYV, batches of mosquitoes were analyzed 7 and 14 days after infection (dpi). From each blood-fed mosquito we collected and assayed two samples for MAYV: head + thorax and saliva. Mixed head and thorax samples were assayed for virus to discriminate infection, and the infection rate was defined as the number of samples with detectable virus divided by all samples tested. Samples of nanoinjected saliva were assayed for virus to discriminate transmission, and the transmission rate was defined as the number of saliva samples with detectable virus divided by all samples from mosquitoes with detectably virus in the body (head + thorax). For infection, 5-day-old adult females were allowed to ingest a mixture of viral supernatant and human blood (2:1). On the first replicate, the viral titer was 1×10^9^ PFU/mL, and for the second, 6×10^9^ PFU/mL, which was offered for 45 minutes through glass feeders using pig intestine as the membrane and a water jacket system with the temperature maintained at 38 °C. Immediately after feeding, fully engorged females were separated and maintained with a 10% sucrose solution until the end of the experiment.

Mosquitoes were anesthetized with CO_2_ and kept on an ice plate while the legs and wings were removed. Each mosquito proboscis was inserted in a 10 μL pipette tip containing a 1:1 solution of 10 μL of 30% sucrose and sterile fetal calf serum. After 30 minutes, the contents of the tips were individually collected in 0.6 mL tubes and stored at −80°C until processing.

In order to test the arboviruses absence in the blood, prior to the experiments, we performed tests for Yellow Fever (YFV), DENV, ZIKV, CHIKV, and MAYV. A group of mosquitoes was fed on the blood sample and, after 10 days post-feeding, these mosquitoes were checked by RT-qPCR. This blood test is a routine procedure in our laboratory to ensure the absence of virus in the blood.

### Confirmation of infectious particles in saliva by nanoinjection

To confirm infectivity in saliva, individual samples of undiluted saliva from each mosquito species were nanoinjected into 10–15 naïve *Ae*. *aegypti* (mosquitoes that had never had contact with any viruses), using a Nanoject II (Drummond Sci) portable injector. In each mosquito, a 276 nL dose of saliva was nanoinjected intrathoracically (pleural membrane) with a pulled glass capillary. Nanoinjected mosquitoes were collected at 5 days post nanoinjection and, on average, 6 whole mosquitoes, per injected saliva, were processed and analyzed by RT-qPCR.

### Virus serial dilution and nanoinjection

To try understanding our findings about the fact some negative mosquito produced positive mosquitoes after saliva nanoinjection (see previous section), we tested the sensitivity of virus detection particles through RT-qPCR. Serial dilutions (10-fold) of known virus stocks were nanoinjected into naïve mosquitoes. Replicates A and B had initial concentrations of 2.09×10^4^ and 5.59×10^4^ viral copies for virus stocks, respectively, and were further diluted 6 times. As a control, uninfected cell supernatant was used. Nanoinjected mosquitoes were collected at 5 days post-nanoinjection and only 5 mosquitoes (whole body), per dilution, were processed and analyzed by RT-qPCR.

### Analysis of MAYV by RT-qPCR

Total RNA from mosquitoes was extracted to verify the infection rates (mosquito head+thorax), transmission rates (through saliva nanoinjection into naïve mosquitoes) or serial virus dilution and nanoinjection. The extraction of RNA viral was performed with the High Pure Viral Nucleic Acid Kit (Roche), following the manufacturer's instructions. The thermocycling conditions were as follows: reverse transcription at 50°C for 10 min, RT inactivation/initial denaturation at 95°C for 30s, 40 cycles of 95°C for 5 s and 60°C for 30s, followed by cooling at 37°C for 30s. The volume of the reaction was 10 μL (5x LightCycler Multiplex RNA Virus Master (Roche), with 1 μM primers, 0.2 μM probe, and 125 ng RNA.

MAYV infection/transmission rates in mosquitoes was quantified by RT-qPCR using a LightCycler 96 (Roche). A multiplex assay was performed according to previous studies, with the MAYV primers MAYVF 5′-GTG GTC GCA CAG TGA ATC TTT C-3′ and MAYVR 5′-CAA ATG TCC ACC AGG CGA AG-3 and the May-Probe 5′-FAM/ATG GTG GTA GGC TAT CCG ACA GGT C/3lABkFQ-3′ (14). For the mosquito control we used the ribosomal gene S17 (RPS17) primers 17S-F 5′-TCC GTG GTA TCT CCA TCA AGC T-3′ and 17S-R 5′-CAC TTC CGG CAC GTA GTT GTC-3′ and the probe 5′-HEX/CAG GAG GAG GAA CGT GAG CGC AG/3BHQ2-3’ (33). All samples were tested in duplicate for MAYV, and the viral genome was determined by comparison with a standard curve using serial dilutions of the target gene cloned into the pGEMT-Easy plasmid (Promega).

## Data analysis

Mosquito infection rate was analyzed with both Person omnibus normality and D’Agostino tests. Fisher’s exact test was then used to assess differences in viral prevalence. Comparisons were significant for *P* values lower than 0.05, and viral load data were compared through the Mann-Whitney U test. All analyses were performed by using Prism V 7.4 (GraphPad).

## Results

### MAYV infection in *Ae*. *aegypti*, *Ae*. *albopictus*, and *Cx*. *quinquefasciatus*

**Fig 1:**
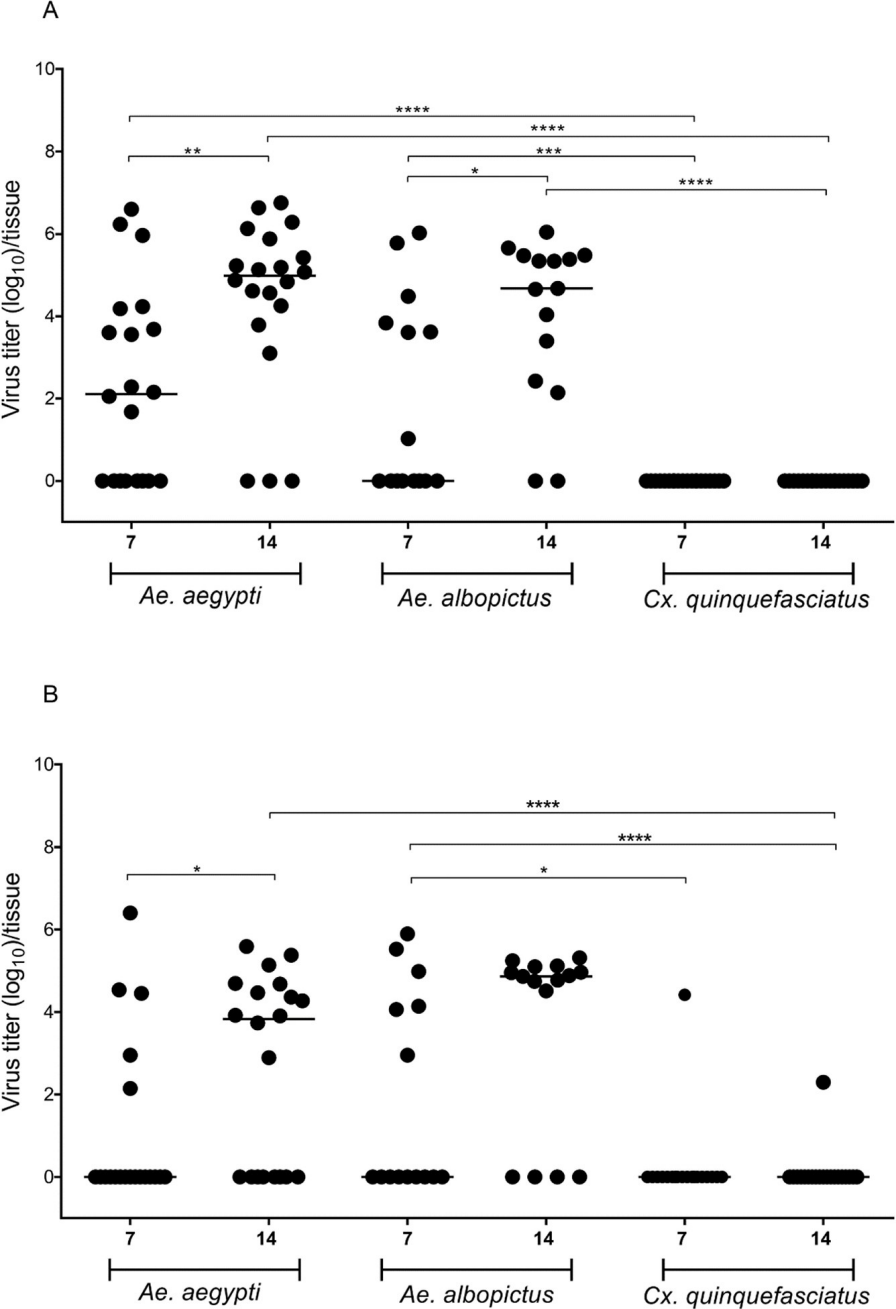
Mosquito viral infection rate (replicates A and B). Each point represents a single head+thorax of adult female, and the black lines indicate the median copy number of the Mayaro virus in each group. The viral titer in the infective blood meal was 1×10^9^ PFU/mL and, and 6×10^9^ PFU/mL respectively for the replicates A and B. The asterisks represent P < 0.05 after the Mann-Whitney U-Test.

Our analysis of different post-infection time points showed that *Ae*. *aegypti* and *Ae*. *albopictus* became positive for MAYV. In addition, in both replicates (Fig 1A and 1B) there was increased infection rate at 14 dpi compared with 7 dpi in *Ae*. *aegypti* and *Ae*. *albopictus*. On the other hand, *Cx*. *quinquefasciatus* mosquitoes were shown to be refractory to MAYV.

In replicate A (Fig 1) at 7 dpi, we observed differences in the infection rate between *Cx*. *quinquefasciatus* and *Ae*. *aegypti* (*p* < 0.0001) or *Ae*. *albopictus* (*p* = 0.0010), but no difference was observed between *Ae*. *aegypti* and *Ae*. *albopictus*. Considering the infection rate at 14 dpi, the same pattern was observed: *Cx*. *quinquefasciatus* was significantly different from *Ae*. *aegypti* (*p* < 0.0001) and *Ae*. *albopictus* (*p* < 0.0001). Comparisons at 7 and 14 dpi for the same mosquito species showed differences for *Ae*. *aegypti* (*p* = 0.0043) and *Ae*. *albopictus* (*p* = 0.0230), clearly showing increasing infection over time.

For replicate B (Fig 1) at 7 dpi, we only observed differences in the infection rate between *Cx*. *quinquefasciatus* and *Ae*. *albopictus* (*p* = 0.0207). At 14 dpi, we observed significantly different infection rate between *Cx*. *quinquefasciatus* and both *Ae*. *aegypti* (*p* < 0.0001) and *Ae*. *albopictus* (*p* < 0.0001). When we compared infection rates at the two time points (7 and 14 dpi), the only mosquito species that showed a difference was *Ae*. *aegypti* (*p* = 0.0262); however, a nonsignificant increasing infection rate trend was observed for *Ae*. *albopictus*.

In general, our combined results from both 7 and 14 dpi show higher susceptibility to MAYV in *Ae*. *albopictus* (61.6%), followed by *Ae*. *aegypti* (57.5%). However, *Cx*. *quinquefasciatus* mosquitoes may be considered refractory to MAYV infection (with only a 2.5% infection rate). The infection rates of MAYV, in each species, in both experiments, and at different dpi are shown in Table 1.

**Table 1:** *Aedes aegypti*, *Aedes albopictus*, and *Culex quinquefasciatus* orally infected with Mayaro virus. The initial viral titer was determined by plaque-forming units (PFU/mL). Infected/total mosquito numbers are shown parenthesis.

MAYV	Titer (PFU/mL)	Days post- infection	*Ae*. *aegypti*	*Ae*. *albopictus*	*Cx*. *quinquefasciatus*
			Infection rate %		
Replicate A	1×10^9^	7	60 (12/20)	46.6 (7/15)	0 (0/20)
		14	85 (17/20)	86.6 (13/15)	0 (0/20)
Replicate B	6×10^9^	7	25 (5/20)	40 (6/15)	5 (1/20)
		14	60 (12/20)	73.3 (11/15)	5 (1/20)

### Amplification of MAYV in mosquitoes after nanoinjection

To verify the occurrence of MAYV in mosquito saliva, we added a virus amplification step in live *Ae*. *aegypti*. Using preamplification by nanoinjection in mosquitoes, MAYV virus was detected in 69.5% (n = 69) of the individual saliva samples from orally infected *Ae*. *aegypti* with detectable amount of virus in their bodies at days 7 and 14 after infection. Likewise, for *Ae*. *albopictus* 71.1% (n = 59) of the mosquitoes with detectable amounts of virus in their bodies also expelled virus in saliva. These results show that orally infected *Ae*. *aegypti* and *Ae*. *albopictus* from Brazil could transmit the virus in their saliva and thus are competent laboratory vectors of MAYV.

**Fig 2:**
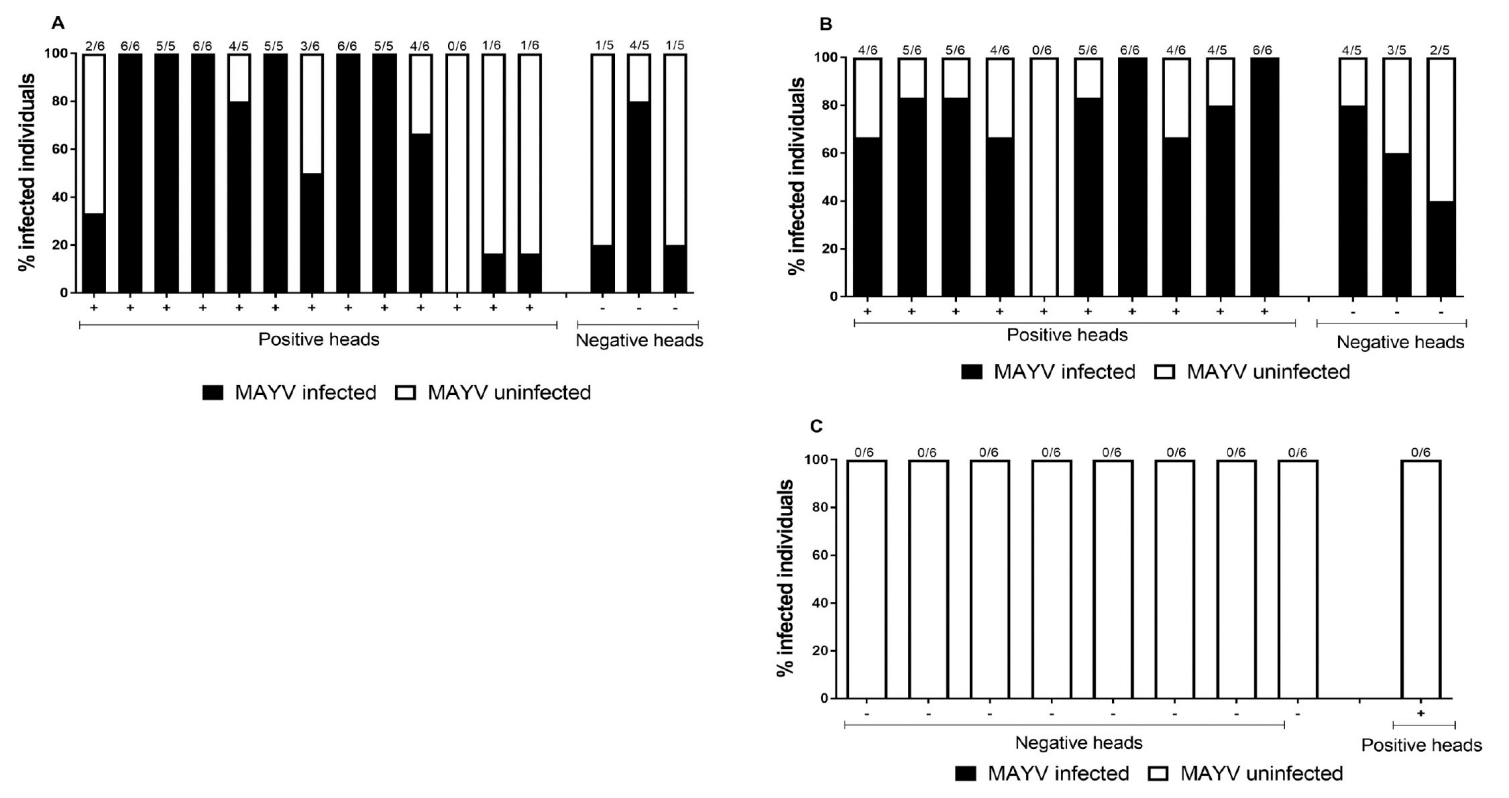
Nanoinjection of saliva from three infected mosquito species into naïve *Aedes aegypti* mosquitoes. Saliva samples were collected from *Aedes aegypti* (A), *Aedes albopictus* (B), and *Culex quinquefasciatus* (C), which were previously infected with MAYV (at 14 dpi), followed by injection into naïve mosquitoes. Mosquitoes that became infected are shown in black and uninfected are depicted in white. Each bar represents a single saliva sample, and the number of transmission rate mosquitoes nanoinjected mosquitoes is given at the top of each bar.

In contrast, none of the 54 nanoinjected mosquitoes with *Cx*. *quinquefasciatus* saliva were able to become infected. We also nanoinjected saliva from negative *Ae*. *aegypti* and *Ae*. *albopictus* mosquitoes (through RT-qPCR) and, surprisingly, some samples were able to infect naïve mosquitoes. Saliva samples from 3 negative *Ae*. *aegypti* (Fig 2A) were nanoinjected into 15 mosquitoes, and 6 (40%) became infected with MAYV. Saliva from 3 negative *Ae*. *albopictus* (Fig 2B) was nanoinjected in 15 mosquitoes, and 9 (60%) became infected with MAYV.

### Virus serial dilution and nanoinjection

In order to try to understand why negative mosquitoes (through RT-qPCR) are able to produce infectious saliva, we performed a series of virus dilutions and injections into naïve mosquitoes, followed by RT-qPCR detection. We found that some viral samples, upon dilution, are not detected through RT-qPCR but are able to infect naïve mosquitoes, using the nanoinjection methodology. Our results showed that RT-qPCR had a detection limit of around 10 copies of the MAYV genome.

## Discussion

To our knowledge, this is the first study to examine the vector competence of *Ae*. *aegypti*, *Ae*. *albopictus*, and *Cx*. *quinquefasciatus* mosquitoes for MAYV (genotype D) in Brazil. Our results show that *Ae*. *aegypti* mosquitoes can become infected with MAYV, and in general, these mosquitoes present high infection rates at 14 dpi, averaging 9.6×10^4^ to 4.7×10^4^ viral genome copies per mosquito for replicates A and B, respectively (Fig 1).

Burstolin *et al*., 2018 [38] evaluated two strains of MAYV; the genotype L strain isolated from *Hg*. *janthinomys* mosquitoes in Para, Brazil, in March 1991, and the genotype D strain originally isolated from a monkey in Para, Brazil, in May 1978. These authors observed in *Ae*. *aegypti* that the genotype L exhibited significantly higher infection rates (86.2% 7 dpi and 51.7% 14 dpi) when compared with the genotype D strain (7.1% at 7 dpi and 0% at 14 dpi). In the present study, using the genotype D strain, we observed higher infection rates (Fig 1 replicates A and B together): 42.5% at 7 dpi and 72.5% at 14 dpi. However, we have to take into consideration that the genetic background of the vector can strongly influence its susceptibility to the virus [39], as well as the relationship between viral titer and infection rate. When we try to look at the relationship between viral titer and percentage of infection rate, we see that Diop *et al*., 2019 [40] using MAYV genotype L with a viral titer of 1×10^6^ focus forming unit (FFU/mL) obtained 53.8% infection rate in *Ae*. *aegypti*, which was like our infection rate (57.5%). Burstolin *et al*., 2018 [38] with a viral titer of 1×10^7^ FFU/mL obtained an infection rate of 86.2% (on 7dpi) and 51.7% (14 dpi) in *Ae*. *aegypti*. Yet, in our experiments, even with a viral titer 2 logs above, we obtained 60% infection rate at 7dpi and 85% at 14dpi for replicate A and 25% at 7dpi and 60% at 14dpi for replicate B (Fig 1).

Our results showed an increasing infection rate over time and overall lower infection rate compared to Burstolin *et al*., 2018 [38], which even with lower viral titer obtained more infected mosquitoes, but with decreasing infection rate over time. Furthermore, these authors noted significantly higher titers in mosquito bodies (7 and 14 dpi), which in most cases were greater than 1×10^6^ viral genome copies per mosquito.

Yet, our results show that although our viral titer is high, fewer mosquitoes passed the range of 1×10^6^ viral genome copies per mosquito. However, we would like to point out that such comparisons are difficult to make since the genotype/isolate and evaluation used by Burstolin *et al*., 2018 [38] and Diop *et al*., 2019 [40] were different from what we used.

A previous study by Pereira *et al*. [41] showed that *Ae*. *aegypti* mosquitoes from Rio de Janeiro were highly susceptible to MAYV, presenting a higher number of viral genome copies per mosquito than those obtained in this study. However, this difference may be related to the viral input at the time of infection or even the genetics of the mosquitoes used in our study. Regarding genetic variability, Gokhale *et al*. [42] suggested that for CHIKV, which is a similar virus to MAYV, the vector genetic background strongly influence the susceptibility to the virus [43].

MAYV cases have been reported in the North, Northeast and Center-West regions of Brazil [21,22,30–33]. *Ae*. *aegypti* mosquitoes naturally infected with MAYV have been found in Cuiabá, Mato Grosso [15], but, at this time, they cannot be incriminated as mosquito species for this virus. Thus, to evaluate viral transmission rate, MAYV-infected saliva of *Ae*. *aegypti* was nanoinjected into naïve mosquitoes, with 69% of mosquitoes becoming infected. Other laboratory studies also confirmed the ability of this vector to transmit MAYV [14,16,41]. Therefore, our results confirm that this mosquito species has great potential for infection/transmission rates of MAYV and, therefore, could play an important role in the transmission of this virus, if it becomes urbanized.

*Ae*. *albopictus* mosquitoes were found to have a similar infection rate to *Ae*. *aegypti*, showing high susceptibility to MAYV. At 14 dpi, significant numbers of viral particles were observed in this species. So far, there are few studies showing the relationship between MAYV and *Ae*. *albopictus*.

Smith and Francy [44], evaluated the vector efficiency of a Brazilian *Ae*. *albopictus* mosquito line fed on viremic hamster blood for MAYV and found that the infection rate ranged from 9% to 16%. The authors classified this strain as being relatively refractory to MAYV infection but suggested that it may become more susceptible, serving as a secondary vector in an outbreak or as a bridge vector between MAYV transmission cycles. Wiggins *et al*. [16] observed in an oral infection experiment that *Ae*. *albopictus* mosquitoes had a significantly higher infection rate than *Ae*. *aegypti* mosquitoes, a similar pattern observed in our study.

Diop *et al*., 2019 [40] using a viral titer of 1×10^6^ FFU/mL (MAYV genotype L) obtained in mosquitoes *Ae*. *albopictus* 76.6% of infection rate. The authors also report the increased expression levels of thioester containing protein 22 (TEP22) and Niemann–Pick type C1 (NPC1- gene responsible for facilitating mosquito infection when infected with Dengue) gene transcripts were observed in infected *Ae*. *albopictus*. In our results, although with a different genotype, we had less infected mosquitoes when fed high viral titer (61.6%—MAYV genotype D), this fact may indicate that for MAYV the viral titer may not be primarily responsible for the success of the infection, but secondary factors such as the viral genotype, the expression of genes related to the mosquito immune system or even the genetic background of the vector.

Infection rates were significantly higher in *Ae*. *albopictus* (85%–100%) than in *Ae*. *aegypti* (67–82%). The same mosquito species may present differentiated vector competence at different sites, since different genotype/genotype interactions between the virus and vector may occur. As an example, it has been demonstrated that the re-emergence of the CHIKV may have been facilitated by the genetic adaptation of the virus to the *Ae*. *albopictus* vector [45,46].

In 2016, the first imported case of MAYV in a French citizen was reported in an area where the *Ae*. *albopictus* mosquito is well established [47], thus highlighting the need to better understand the vector competence of this mosquito, as well as its possible role in the transmission of MAYV.

To evaluate viral transmission rate in our experiments, *Ae*. *albopictus* mosquito saliva submitted to MAYV infection was nanoinjected into naïve mosquitoes, resulting in a high rate of infectivity. Smith and Francy [44], noted in their study that approximately half (5/11) of the mosquitoes infected with hamster viremic blood were able to transmit MAYV when their saliva was tested in capillary tubes. Wiggins *et al*. [16] observed that *Ae*. *albopictus* mosquitoes exhibited low transmission rate of MAYV in saliva expectorates. However, these authors used a different methodology.

We also attempted to study whether saliva originating from negative mosquitoes samples were able to infect naïve mosquitoes. Unexpectedly, negative mosquitoes samples from *Ae*. *aegypti* and *Ae*. *albopictus* were able to infect other mosquitoes through their saliva. Previous experiments in our group have shown the same effect for DENV with a negative thorax and positive saliva, but it resulted in a lower infection rate.

Furthermore, some studies have described similar results in *Ae*. *aegypti* and *Cx*. *quinquefasciatus* with ZIKV, DENV, and WNV [48–50]. One important hypothesis to consider is that over the course of infection, viral decline occurs in other tissues, but the salivary glands/saliva remains positive for transmission. Furthermore, we suspect that mosquito salivation may, in some cases, deplete the glands of almost all viral particles, which could not be detected (in the head) through RTq-PCR.

To better understand these findings, we investigated the relationship between the amounts of (detectable) particles required for mosquito infection. It was possible to observe that samples classified as negative for RT-qPCR were able to infect other mosquitoes, indicating that only a few viral particles are necessary to initiate infection. In addition, it was observed that regardless of the nanoinjected viral doses they produced between 5.6×10^5^ to 1.4×10^6^ viral genome copies per mosquito respectively for replicates A and B (S1 Fig). Therefore, nanoinjection of the viral saliva/dilution in the mosquito acts as a model that amplifies the particles and facilitates later detection [51]. This may explain why saliva from heads classified as negative produced positive mosquitoes upon injection. We believe that only a few viral particles, which cannot be detected through RT-qPCR, are required to infect a mosquito. We suggest that for a broader understanding of this finding, complementary studies using immunofluorescence assays to detect transmission through saliva originating from PCR-negative heads should be undertaken.

When evaluating the vector competence of *Cx*. *quinquefasciatus*, only two mosquitoes were found to be positive for MAYV, and no mosquito became infected after injection with saliva from these *Cx*. *quinquefasciatus* mosquitoes submitted to MAYV infection. Corroborating our data, Brustolin *et al*. [38], also tested the vector competence of *Cx*. *quinquefasciatus* and observed that this mosquito had either poor or null infection and transmission rates for MAYV.

*Cx*. *quinquefasciatus* is quite abundant in Brazil, and to date there is only a single record of this mosquito harboring MAYV in Cuiabá [15]. This mosquito is, however, a vector of *Wuchereria bancrofti* in Brazil, an etiologic agent of lymphatic filariasis in humans [52], and was recently incriminated in ZIKV transmission in the metropolitan region of Pernambuco [8]. Guo *et al*. [48], have also demonstrated the vector competence of *Cx*. *quinquefasciatus* for ZIKV in China. In contrast, several other studies have demonstrated the lack of ability of this mosquito to infect and transmit ZIKV [53–55]. These results confirm that the vector competence of the same mosquito species can vary geographically, emphasizing the importance of studying the vector competence of different mosquitoes and from different localities.

In conclusion, our studies show that, under laboratory conditions, *Ae*. *aegypti* and *Ae*. *albopictus* can be infected and potentially transmit MAYV (genotype D), although *Cx*. *quinquefasciatus* exhibited poor vector competence. To our knowledge this is the first report of a study involving the vector competence of a particular MAYV genotype, done simultaneously with three mosquito species. *Ae*. *aegypti* and *Ae*. *albopictus* are widely distributed throughout the Americas [3,56], and although they are not the main vectors for MAYV, they can potentially play a significant role in the transmission rate of this virus. We suggest that further studies should be conducted to demonstrate the vector competence of the same species towards other MAYV genotypes and even isolates, since distinct isolates of the same virus can behave differently on the same vector species. Furthermore, studies to demonstrate the co-infections with other arboviruses, as well as ecological studies on the recent YFY outbreaks, considering that *Hg*. *janthinomys* is a common vector for both viruses.

## Supporting information

S1 Fig Sample dilutions and detection of Mayaro virus. A and B represent different replicates. Each viral dilution (represented by green dots) was nanoinjected into naïve mosquitoes, followed by virus detection through RT-qPCR (red dots). Samples 8A and 8B are mock controls. Each red spot represents a single female mosquito.
(DOCX)
