# Supplementary figures and images for: Vector competence of Aedes aegypti, Aedes albopictus, and Culex quinquefasciatus mosquitoes for Mayaro virus
Source: PLoS Negl Trop Dis. 2020 Apr 14;14(4):e0007518. doi: 10.1371/journal.pntd.0007518 (PMC7182273; doi:10.1371/journal.pntd.0007518)

**Viral dilution and detection of MAYV**
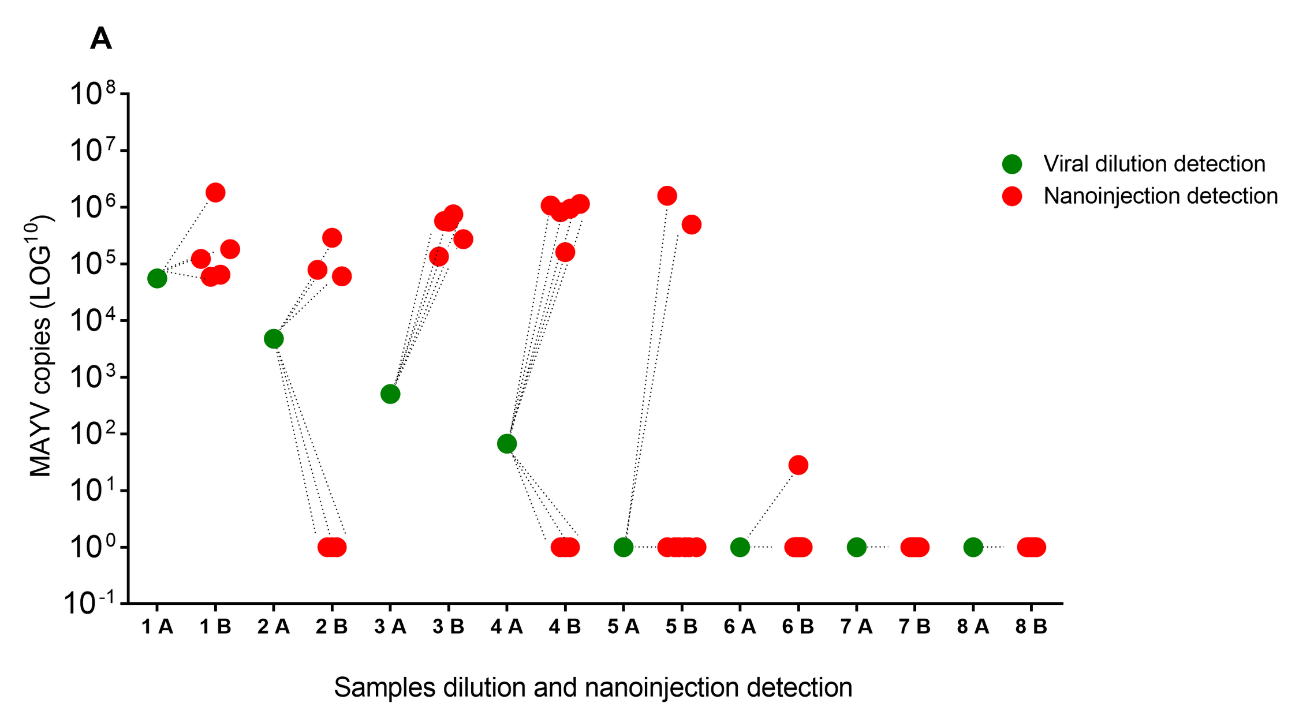


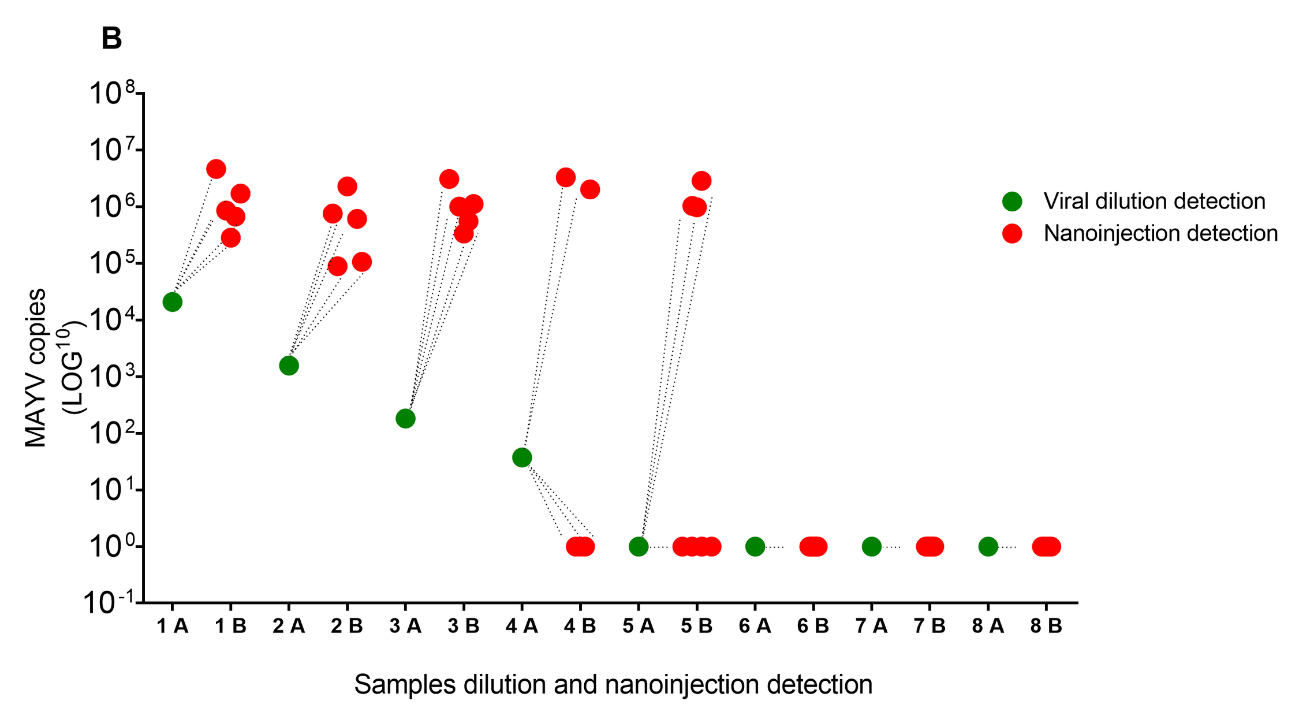

Supplement: S1 Fig — A and B represent different replicates. Each viral dilution (represented by green dots) was nanoinjected into naïve mosquitoes, followed by virus detection through RT-qPCR (red dots). Samples 8A and 8B are mock controls. Each red spot represents a single female mosquito. (DOCX) [file pntd.0007518.s001.docx]
